# Shikonin Attenuates Chronic Cerebral Hypoperfusion-Induced Cognitive Impairment by Inhibiting Apoptosis via PTEN/Akt/CREB/BDNF Signaling

**DOI:** 10.1155/2021/5564246

**Published:** 2021-06-07

**Authors:** Yanqiu Jia, Zhe Li, Tianjun Wang, Mingyue Fan, Jiaxi Song, Peiyuan Lv, Wei Jin

**Affiliations:** Department of Neurology, Hebei General Hospital, Shijiazhuang, China

## Abstract

Shikonin (SK) exerts neuroprotective effects; however, to date, its protective effect against chronic cerebral hypoperfusion- (CCH-) induced vascular dementia (VaD) has not been investigated. Therefore, the current study investigated whether SK could mitigate the cognitive deficits caused by CCH. The effects of SK treatment on the PTEN/Akt/CREB/BDNF signaling pathway and apoptosis in hippocampal neurons were examined in a rat model of VaD established via bilateral common carotid artery occlusion (BCCAO). Fifty-two rats were randomly divided into 4 groups: sham, vehicle, SK-L (10 mg/kg SK per day), and SK-H (25 mg/kg SK per day). SK was regularly administered by gavage for 2 weeks. The results of the water maze test revealed that the escape latency in the vehicle group was significantly longer than that in the sham group, and rats in the vehicle group spent a smaller proportion of time in the target quadrant than those in the sham group. SK treatment reduced the escape latencies and increased the proportion of time spent in the target quadrant. Nissl staining showed morphological damage in the CA1 areas of the hippocampus in the vehicle group. SK treatment alleviated the injuries to hippocampal neurons. Western blot analysis showed higher p-PTEN and lower p-Akt, p-CREB, and BDNF expression in the vehicle group than in the sham group. SK administration reversed the upregulation of p-PTEN and the downregulation of p-Akt, p-CREB, and BDNF. The number of terminal deoxynucleotidyl transferase-mediated dUTP nick-end labeling- (TUNEL-) positive cells in the hippocampal CA1 region of the vehicle group was significantly increased. Treatment with SK decreased the number of positive cells. Furthermore, as marker proteins of apoptosis, bcl-2 expression was decreased and bax expression was increased; thus, the ratio of bcl-2/bax was decreased in the vehicle group. SK treatment upregulated the expression of bcl-2 and downregulated the expression of bax, thereby elevating the bcl-2/bax ratio. Moreover, the aforementioned effects of SK were dose-dependent. The effect of 25 mg/kg per day was more obvious than that of 10 mg/kg per day. In conclusion, SK inhibited hippocampal neuronal apoptosis to protect against CCH-induced injury by regulating the PTEN/Akt/CREB/BDNF signaling pathway, consequently improving cognitive impairment.

## 1. Introduction

With the increasing incidence rate of cerebrovascular disease, vascular dementia (VaD) has become one of the common dementia types in elderly individuals [1]. Diseases that lead to VaD include hemorrhagic cerebrovascular disease, ischemic cerebrovascular disease, and acute and chronic hypoxic cerebrovascular disease [2]. Chronic cerebral hypoperfusion (CCH) has an especially close relationship with the pathogenesis of VaD [1]. However, the mechanism of CCH-induced VaD is still unclear.

Normal brain tissue needs an abundant blood supply to provide adequate energy and nutrition for the survival of nerve cells. Studies have shown that CCH causes ischemia and hypoxia, which activate a series of pathological reactions, including neuroinflammation, oxidative stress, glutamate excitotoxicity, and calcium overload [3–5]. These events interact with each other and activate downstream signaling pathways, leading to neuronal damage through mechanisms such as apoptosis and excessive autophagy [6, 7]. The hippocampal area, which is considered to be closely associated with cognitive function, is sensitive to ischemia [8]. Studies have confirmed that CCH can induce progressive apoptosis in hippocampal neurons, which is considered to be a critical factor in cognitive decline [9].

CCH causes neuronal apoptosis through several cell signaling pathways. Among them, the phospho-PI3 kinase (PI3K)/protein kinase B (Akt) signaling pathway plays an important role in cell survival. As an upstream inhibitor of the PI3K/Akt pathway, phosphatase and tensin homolog deleted on chromosome ten (PTEN) can catalyze the conversion of phosphatidylinositol 3,4,5-triphosphate (PIP3) to phosphatidylinositol 4,5-diphosphate (PIP2) via inositol-3 phosphatase activity and then negatively regulate the activity of Akt. PTEN is highly expressed in the nervous system and has received increasing attention in recent years [10]. Previous studies have shown that PTEN participates in CCH-induced cognitive impairment. However, there are still controversies [11–14], which have aroused our interest.

The root of the perennial plant *Lithospermum erythrorhizon* has been used in traditional Chinese medicine for many years. One of its extracts, shikonin (SK; molecular formula: C_16_H_16_O_5_), has been proven to be effective in various types of cancers [15]. Treatment with SK leads to necrosis, apoptosis, or autophagy in cancer cells through a complex signaling network, thus inhibiting cancer cell proliferation, metastasis, and invasion [16, 17]. Unlike cancer cells, neurons are highly differentiated; thus, SK treatment may affect these cells differently [18]. Studies have shown that SK has antiapoptotic effects against ischemic damage [18, 19] and spinal cord injury [20]. Pretreatment with SK can improve the cognitive function of carbon ion beam-irradiated mice [21].

However, no data are currently available on the possible protective effect of SK on CCH-induced VaD. Hence, in the present study, a classic VaD model induced by bilateral common carotid artery occlusion (BCCAO) in rats [22, 23] was used to observe whether SK could improve CCH-induced cognitive deficits by inhibiting apoptosis in hippocampal neurons. Furthermore, we investigated whether the neuroprotective effects were achieved via the PTEN/Akt/cAMP-response element binding protein (CREB)/brain-derived neurotrophic factor (BDNF) pathway.

## 2. Materials and Methods

### 2.1. Experimental Animals and Drugs

Fifty-two male Sprague-Dawley rats (clean grade, aged 3 months, weighing 250–300 g) were purchased from the Hebei Medical University Experimental Animal Center and housed in the Hebei General Hospital Animal Center for 1 week with food and water *ad libitum*. The animal center was kept at a temperature of 22–24°C with a 12 h light/dark cycle using natural light. Protocols were approved by the Animal Care and Management Committee of Hebei General Hospital (SCXK2016-0006) and were in line with the regulations of laboratory animal management defined by the Ministry of Science and Technology of the People's Republic of China (1988) no. 134. All efforts were made to minimize animal suffering and reduce the number of animals used. SK (Santa Cruz Biotechnology) was dissolved in dimethyl sulfoxide (DMSO).

The VaD rat model was established by BCCAO (method detailed below). The rats were randomly assigned to one of 4 groups: the sham-operated control group (sham; *n* = 13), which received equal volumes of PBS + 1% DMSO daily; the vehicle group (vehicle; *n* = 13), which received equal volumes of PBS + 1% DMSO daily after BCCAO; the low-dose group (SK-L; *n* = 13), which was treated with 10 mg/kg SK per day after BCCAO; and the high-dose group (SK–H; *n* = 13), which was treated with 25 mg/kg SK per day after BCCAO. The drug/vehicle was regularly administered by gavage for 2 weeks.

### 2.2. BCCAO Protocol

The classic VaD model induced by BCCAO was used to induce permanent CCH. Animals were fasted for 12 h before surgery but were freely allowed to drink water. The rats were anesthetized with 2% sodium pentobarbital (50 mg/kg body weight, i.p.). A ventral cervical incision was made to expose the bilateral common carotid artery, which was then separated from the carotid sheath and vagus nerve. In the vehicle and drug treatment (SK-L and SK-H) groups, the common carotid arteries were double ligated with 4–0 silk sutures. To ensure that arterial blood flow was blocked, the arteries were cut between the ligations. The sham group underwent the same procedure excluding the ligations. During the procedure, an incandescent lamp was used to maintain a body temperature of 36.5–37.5°C.

### 2.3. Behavioral Tests

The Morris water maze (MWM; Shanghai Jiliang Software Technology Co., Ltd., Shanghai, China) task was used to behaviorally assess learning and memory in the experimental groups. The test was initiated on postoperative day 15 and lasted 6 days. The MWM consists of a black, circular pool (diameter: 160 cm; depth: 45 cm), conceptually divided into 4 quadrants and surrounded by reference objects with different colors and shapes in fixed positions. For the first phase of the task (the place navigation test), a black circular platform with a diameter of 10 cm was placed in the middle of a selected quadrant (the target quadrant) and submerged approximately 2 cm below the water surface. An automatic temperature control system kept the water at 22–23°C. The day before the test, the rats were allowed to swim freely for 2 min to adapt to the water maze environment without a platform. On the following day, the platform was placed in the water maze. A random quadrant was selected as the entry point, and the rats were gently placed into the water facing the wall. A camera system connected to a tracking device was positioned above the water maze. This camera was used to record swimming trajectory data, which were then transferred to an image analyzer. The tracking software automatically recorded the escape latency, the time spent between entering the water and climbing onto the submerged platform. If the rats failed to find the platform within 120 s, they were guided onto the platform and allowed to rest on the platform for 10 s. In these cases, the escape latencies were recorded as 120 s. Training was performed 4 times per day for 5 consecutive days. On the sixth day of training, the rats entered the second phase of the task: the spatial probe test. For this test, the platform was removed, and the quadrant opposite to the target quadrant was selected as the entry point. Rats were allowed to swim freely for 120 s. The percentage of the total swimming time that each rat spent in the target quadrant was recorded.

### 2.4. Nissl Staining

After the MWM test, six rats were selected randomly from each group, deeply anesthetized by 2% sodium pentobarbital (50 mg/kg, i.p.), and rapidly perfused with 100 ml of 0.9% saline followed by 400 ml of 4% paraformaldehyde in 0.1 M phosphate buffer (pH 7.4) through the ascending aorta. The rat brains were then embedded in paraffin. After fixation, the brains were coronally sectioned at a thickness of 5 *μ*m and stained with cresyl violet. All stained sections were photographed and examined with a microscope (Olympus, Japan).

### 2.5. Terminal Deoxynucleotidyl Transferase-Mediated dUTP Nick-End Labeling (TUNEL)

Histological sections were processed using the TUNEL assay to label apoptotic cells (In Situ Cell Death Detection Kit, Roche Applied Science, Mannheim, Germany) according to the manufacturer's protocol. The number of TUNEL-positive neurons in three visual fields (400x) for each rat was counted using Image-Pro Plus software 5.0 (Media Cybernetics, USA). The average value was used as statistical data.

### 2.6. Western Blotting

Following the water maze test, 6 rats were randomly selected from each group for western blot analysis. After deep anesthesia (2% sodium pentobarbital, 50 mg/kg, i.p.), the bilateral hippocampi were removed, placed on ice, and then homogenized in radioimmunoprecipitation assay buffer containing phenylmethylsulfonyl fluoride (BioTeke Corporation, Beijing, China). After being centrifuged at 12,000 rpm for 10 min at 4°C, the protein concentration of the supernatant was determined using the bicinchoninic acid method (Thermo-Fisher Scientific, USA), and loading buffer was then added for heated denaturation. Next, sodium dodecyl sulfate-polyacrylamide gel electrophoresis (SDS-PAGE) was performed, and the proteins were transferred to a polyvinylidene difluoride (PVDF) membrane. The PVDF membrane was blocked with 5% nonfat milk dissolved in Tris-buffered saline and Tween-20 (TBST) for 2 h at room temperature and then incubated overnight at 4°C with the following primary antibodies: anti-*β*-actin (1 : 500; Proteintech, Chicago, IL, USA), anti-P-PTEN (Ser380) (1 : 1,000; Cell Signaling Technology, MA, USA), anti-Akt (1 : 1,000; Abcam, Cambridge, MA, USA), anti-P-Akt (Ser473) (1 : 1,000; Abcam, Cambridge, MA, UK), anti-CREB (1 : 800; Abcam, Cambridge, MA, UK), anti-P-CREB (Ser133) (1 : 600; Abcam, Cambridge, MA, UK), anti-BDNF (1 : 800; Abcam, Cambridge, MA, UK), anti-Bax (1 : 1,000; Abcam, Cambridge, MA, UK), and anti-Bcl-2 (1 : 1,000; Abcam, Cambridge, MA, UK). The next day, the PVDF membrane was washed with TBST (3 washes, 10 min each) and then incubated with horseradish peroxidase-labeled goat anti-rabbit or goat anti-rat secondary antibodies (1 : 10,000; SAB, USA) in TBST at room temperature for 1–2 h. The membranes were washed with TBST (3 washes, 10 min each). The enhanced chemiluminescence (ECL) reaction was used to detect protein bands on the PVDF membranes, and images were analyzed using the image analysis software ImageJ (version 1.30v; Wayne Rasband, National Institutes of Health, Bethesda, MD). The relative intensities of the protein bands were normalized to *β*-actin.

### 2.7. Statistical Analysis

SPSS 21.0 software was used to analyze the quantitative data in the study, which are expressed as the mean ± standard deviation (SD). The escape latencies were analyzed with repeated measures analysis of variance (ANOVA). All other data analyses were performed using one-way ANOVA. Post-hoc intergroup comparisons were conducted using the Student–Newman–Keuls (SNK) test. Values of *p* < 0.05 were considered to be statistically significant.

## 3. Results

### 3.1. BCCAO-Induced Spatial Learning and Memory Impairments Were Ameliorated by SK Treatment

**Figure 1:**
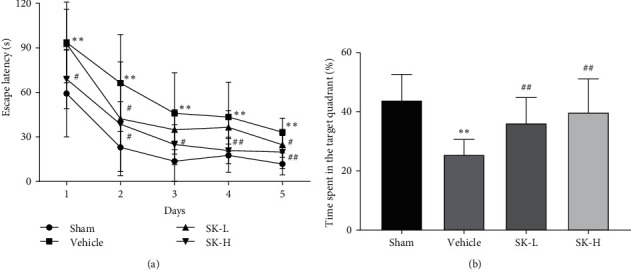
Effect of SK on BCCAO-induced spatial learning and memory impairment in rats as measured by the Morris water maze test. Values are expressed as mean ± SD. (a) Changes in daily escape latencies to find the hidden platform at week 2 after BCCAO.  ^*∗*^*p* < 0.01 vs. sham group;  ^#^*p* < 0.01 vs. vehicle group. (b) Percentage of time spent in the target quadrant during the probe trial at week 2 after BCCAO.  ^*∗*^*p* < 0.05 vs. sham group;  ^#^*p* < 0.01 vs. vehicle group.

In the Morris water maze test, escape latencies gradually shortened. On days one to five, the escape latency of the vehicle group was significantly longer than that of the sham group (*p* < 0.01). Compared with the vehicle group, both the SK-L and SK-H groups had shorter escape latencies during the five days (*p* < 0.01 for both groups; Figure 1(a)). The ratio of the time spent in the target quadrant in the absence of a submerged platform was significantly different between the groups. As shown in Figure 1(b), compared with rats in the sham group, rats in the vehicle group spent a significantly smaller proportion of time in the target quadrant (*p* < 0.01), while rats in the SK-L and SK-H groups spent larger proportions of time in the target quadrant (*p* < 0.01).

### 3.2. Morphological Reorganization Was Observed in the Hippocampus of SK-Treated BCCAO Rats

**Figure 2:**
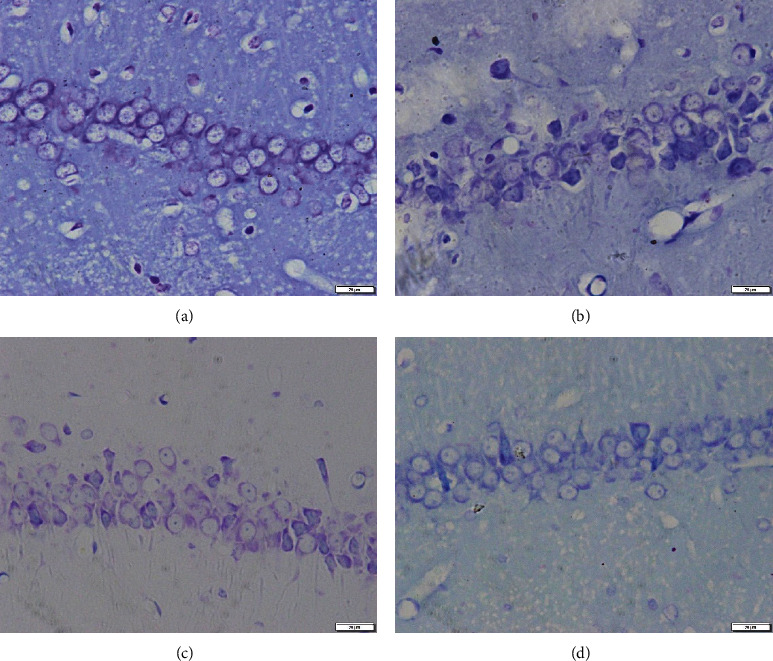
Nissl staining showing the effects of SK on neuronal morphology in hippocampal area CA1. Representative images of pyramidal neuron damage in the CA1 region. Scale bar = 20 *μ*m. Magnification: 400x. (a) Sham group; (b) vehicle group; (c) SK-L group; (d) SK-H group.

The pyramidal cells in the CA1 areas of the hippocampus in the sham group were hierarchically and evenly arranged, with complete structures, uniform distribution of chromatin, large and round nuclei, and clear nucleoli and cytoplasms. In contrast, neurons in the vehicle group were loosely and irregularly arranged, with some neurons showing significant shrinkage, as well as darkly stained and condensed nuclei. As shown in Figures 2(a)–2(d), the neuronal morphological changes observed in the vehicle group were partially reversed in the SK treatment groups, especially in the SK-H group.

### 3.3. SK Reduced the Number of TUNEL-Positive Cells in the CA1 Region of the Hippocampus

**Figure 3:**
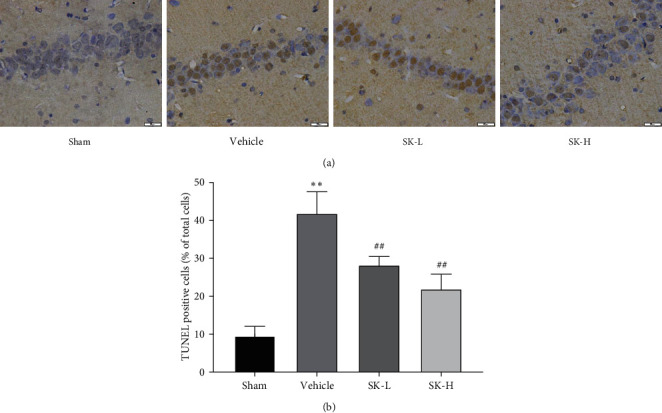
(a) TUNEL staining shows apoptotic cells in the CA1 region of hippocampus. Scale bar = 20 *μ*m. Magnification: 400x. (b) Quantitative analysis of the data from TUNEL staining (*n* = 6) (mean ± SD, *n* = 6 in each group). ^*∗∗*^*p* < 0.01 vs. sham group;  ^##^*p* < 0.01 vs. vehicle group.

The TUNEL staining results are shown in Figure 3.  Figure 3(a) shows representative images of TUNEL staining. Compared with that in the sham group, the number of positive cells in the model group was significantly increased (*p* < 0.01); compared with the vehicle group, treatment with SK (10 mg/kg per day and 25 mg/kg per day) decreased the number of positive cells (*p* < 0.01) (Figure 3(b)).

### 3.4. SK Upregulated Bcl-2 and Downregulated Bax in the Hippocampus of BCCAO Rats

**Figure 4:**
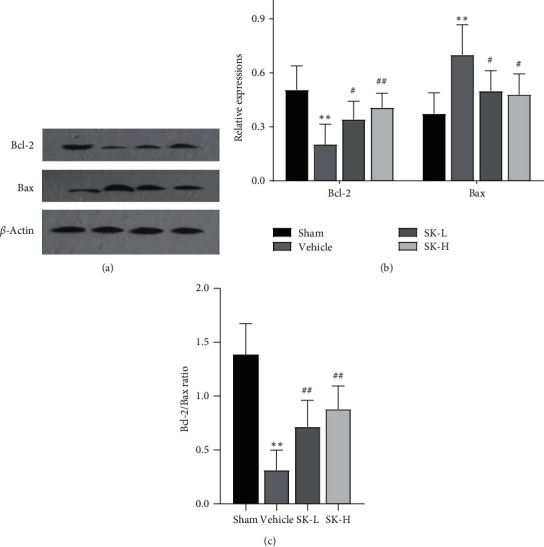
(a) Representative western blots of Bcl-2 and Bax. *β*-actin served as the control for protein loading. (b) Quantitative analysis of Bcl-2 and Bax proteins. (c) The ratio of Bcl-2/Bax (mean ± SD, *n* = 6 in each group). ^*∗∗*^*p* < 0.01 vs. sham group;  ^#^*p* < 0.05,  ^##^*p* < 0.01 vs. vehicle group.

To evaluate the level of hippocampal neuronal apoptosis, the expression of bcl-2 and bax, two key markers of apoptosis, was analyzed.  Figure 4(a) shows representative western blots of Bcl-2 and Bax expression. Significant differences in bcl-2 and bax expression were found in all groups (F (3,20) = 8.390, *p* < 0.01; F (3,20) = 6.633, *p* < 0.01, respectively) (Figure 4(b)). The bcl-2/bax ratio was different among all groups (F (3,20) = 21.003, *p* < 0.01) (Figure 4(c)). Compared with the sham group, the vehicle group showed a significant decrease in bcl-2 expression (*p* < 0.01) and an increase in bax expression (*p* < 0.01); thus, the bcl-2/bax ratio was significantly smaller in the vehicle group than in the sham group (*p* < 0.01) (Figures 4(b) and 4(c)). Treatment with SK (10 mg/kg per day and 25 mg/kg per day) was associated with an increase in bcl-2 expression levels (*p* < 0.05, *p* < 0.01, respectively) and a decrease in the expression of bax (*p* < 0.05), thereby increasing the bcl-2/bax ratio (*p* < 0.01) (Figures 4(b) and 4(c)).

### 3.5. SK Downregulated the Expression of p-PTEN and Upregulated the Expression of p-Akt, p-CREB, and BDNF in the Hippocampus of BCCAO Rats

**Figure 5:**
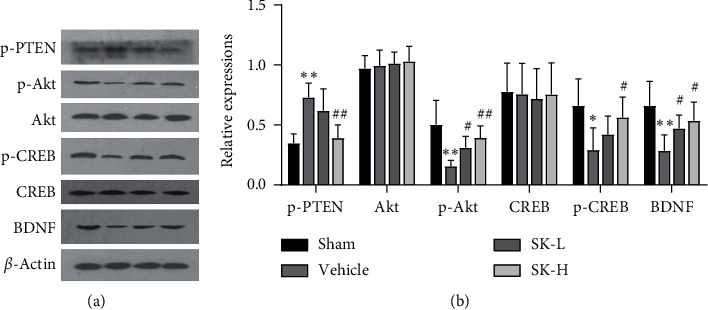
(a) Representative western blots of p-PTEN, Akt, p-Akt, CREB, p-CREB, and BDNF. *β*-actin served as the control for protein loading. (b) Quantitative analysis of the western blot data (*n* = 6) (mean ± SD, *n* = 6 in each group).  ^*∗*^*p* < 0.05, ^*∗∗*^*p* < 0.01 vs. sham group;  ^#^*p* < 0.05,  ^##^*p* < 0.01 vs. vehicle group.

To determine whether the PTEN/Akt/CREB/BDNF signaling pathway participates in the neuroprotective effect of SK, the protein levels of p-PTEN, p-Akt, Akt, p-CREB, CREB, and BDNF were analyzed by western blotting (representative western blots are shown in Figure 5(a)). Statistical analysis showed significant differences in p-PTEN, p-Akt, p-CREB, and BDNF expression between the tested groups (F (3,20) = 12.253, *p* < 0.01; F (3,20) = 8.421, *p* < 0.01; F (3,20) = 4.574, *p* < 0.05; F (3,20) = 6.143, *p* < 0.01, respectively). However, there were no significant differences in Akt or CREB expression between the groups (F (3,20) = 0.271, *p* > 0.05; F (3,20) = 0.057, *p* > 0.05, respectively). In the post-hoc SNK test, the expression of p-PTEN in the vehicle group was higher than that in the sham group, while the expression of p-Akt, p-CREB, and BDNF in the vehicle group was lower than that in the sham group (*p* < 0.01, *p* < 0.01, *p* < 0.05, and *p* < 0.01, respectively). The administration of SK (10 mg/kg per day and 25 mg/kg per day) reversed the upregulation of p-PTEN (*p* > 0.05 and *p* < 0.01, respectively) and the downregulation of p-Akt (*p* < 0.05 and *p* < 0.01, respectively), p-CREB (*p* > 0.05 and *p* < 0.05, respectively), and BDNF (*p* < 0.05) (Figure 5(b)).

## 4. Discussion

VaD is a preventable type of dementia, and early intervention can partly improve disease prognosis [24]. Due to the complex mechanisms underlying VaD, the development of therapeutic strategies is needed [25, 26].

Based on its ability to induce apoptosis in tumor tissue, SK has been widely investigated in cancer treatment [27–30]. In recent years, the antiapoptotic effects of SK have been gradually discovered in lung injury [31], myocardial ischemic/reperfusion injury [32], osteoarthritis [33], and nervous system diseases [18, 20, 34].

Notably, by regulating inflammatory responses, ameliorating blood-brain barrier permeability [19], and reducing apoptosis [18], SK has been shown to effectively protect against ischemic damage. However, to date, there have been no reports about the effect of SK on cognitive impairment induced by CCH. Can SK improve the cognitive decline induced by CCH through its antiapoptotic effect? We explored this question in the current study.

By using BCCAO rats, a widely recognized classic CCH-induced VaD model [23], we found that SK could improve the spatial learning and memory abilities of CCH rats, suggesting that SK may have a therapeutic effect against VaD.

Persistent neuronal apoptosis may underlie the progressive cognitive dysfunction associated with CCH [7]. The Bcl-2 family is one of the most important gene families that regulate apoptosis. Bax is located in the outer membrane of mitochondria and is involved in homeostasis in nonapoptotic cells. When cells are stimulated by stress, bax relocates and undergoes conformational changes that induce mitochondrial perforation, thereby initiating apoptosis. Upregulated bcl-2 can form a dimer with bax, which inhibits apoptosis [35]. The present findings indicate that SK can regulate the expression of bcl-2 and bax, thereby inhibiting CCH-induced apoptosis in hippocampal cells, which was consistent with the TUNEL results.

As a key signaling pathway that regulates apoptosis, the PTEN/Akt signaling pathway and downstream CREB/BDNF were analyzed by western blotting. PTEN, a tumor suppressor gene with bispecific phosphatase activity, is closely associated with tumorigenesis, similar to the p53 gene. PTEN is highly expressed in brain tissues [10], and its activity is regulated through phosphorylation, ubiquitination, receptor binding, and subcellular localization [36, 37]. Evidence has shown that PTEN dysfunction not only contributes to tumorigenesis in the central nervous system but also participates in the pathogenesis of brain ischemia and neurological and mental disorders [38]. PTEN may also be involved in injury protection and regeneration in the central nervous system [39]. Regarding the phosphorylation level of PTEN after CCH, there is still controversy among related studies [11–14]. In the present study, we found that p-PTEN was increased in the hippocampus after 2 weeks of CCH. This change was consistent with three previous studies [12–14], in which the durations of CCH were 2 weeks, 3 weeks, and 4 weeks. However, Chen et al. reported a decrease in the p-PTEN level in the same animal model after 8 weeks of hypoperfusion [11]. We suspect that dynamic changes in p-PTEN exist in different stages of CCH.

In theory, as an upstream inhibitor of Akt, PTEN is inactivated after phosphorylation at Ser380, thereby increasing Akt activity [40]. However, we found decreased Akt phosphorylation following elevations in p-PTEN in CCH rats. A possible explanation is that PTEN activity is regulated in multiple ways, such as through ubiquitination or subcellular localization following CCH [41].

Akt is a serine/threonine kinase that mediates various biological effects. Akt participates in cell growth, proliferation, migration, glycometabolism, transcription, protein synthesis, angiogenesis, and cell survival [42, 43]. CREB, an important downstream molecule of Akt, is associated with neuronal survival and axon growth [44]. CREB phosphorylation at the Ser133 site induces its activation [44]. Phosphorylated CREB directly or indirectly activates the transcription of related genes, including c-fos, bcl-2, and some neurotrophic factors, such as basic fibroblast growth factor and BDNF, ultimately affecting cognition [14, 45]. BDNF promotes nerve durability by promoting the expression of the antiapoptotic protein bcl-2 and increasing synaptic plasticity [46, 47]. BDNF also activates the PI3K/Akt signaling pathway and then forms a positive feedback loop to promote cell survival [48, 49].

Previous studies have reported decreases in the phosphorylation levels of Akt and CREB, as well as in the expression of BDNF, in the hippocampus of CCH rats [45, 50, 51], which suggests that this signaling pathway may be involved in the development of cognitive impairment in these rats. In this study, the expression of p-Akt, p-CREB, and BDNF in the vehicle group was lower than that in the sham group, which is consistent with these previous findings. For the first time, we showed that treatment with SK could increase the phosphorylation levels of Akt and CREB and the expression of BDNF, accompanied by antiapoptotic effects, suggesting that SK may reduce apoptosis through this pathway and ultimately exert neuroprotective effects and improve cognition.

Additionally, our findings indicate that treatment with a higher SK dose (25 mg/kg, daily) showed more robust effects on the expression of these proteins than treatment with a lower dose (10 mg/kg, daily), indicating that the therapeutic effects of SK are dose-dependent in a certain dose range.

Our results suggest that SK can improve the cognitive impairment induced by CCH. Of course, this study has certain limitations. First, no specific pathway inhibitors were used in this study; hence, whether the antiapoptotic effect of SK observed herein is PTEN/Akt/CREB/BDNF-dependent remains to be determined. Second, changes in the PTEN/Akt/CREB/BDNF pathway were not analyzed. Multiple time point analysis may be able to better reveal changes in expression to more precisely explain the role of this pathway. Third, only the hippocampus was evaluated to confirm the therapeutic effect of SK, but the cortex and other brain areas were not examined. Further research is needed to solve these problems.

## 5. Conclusion

The findings of the present study indicate that SK may inhibit hippocampal neuron apoptosis and protect against injury induced by CCH by regulating the PTEN/Akt/CREB/BDNF signaling pathway and thereby improve cognitive impairment.

## Data Availability

The data used to support the findings of this study are available from the corresponding author upon request.
